# Validation of virtual simulation content for prevention of unplanned extubation in intensive care*

**DOI:** 10.1590/1980-220X-REEUSP-2024-0443en

**Published:** 2025-06-09

**Authors:** Cinthya Helena dos Anjos Carvalho, Daniela Couto Carvalho Barra, Ana Graziela Alvarez, Neide da Silva Knihs, Grace Teresinha Marcon Dal Sasso, Ricardo João Cruz-Correia, Pedro Miguel Garcez Sardo

**Affiliations:** 1 Universidade Federal de Santa Catarina, Departamento de Enfermagem, Florianópolis, SC, Brazil.; 2 Universidade do Porto, Faculdade de Medicina, Departamento de Medicina Comunitária, Informação e Ciências da Decisão em Saúde, Centro de Investigação em Tecnologias e Serviços de Saúde, Porto, Portugal.; 3 Universidade de Aveiro, Escola Superior de Saúde, Aveiro, Portugal.

**Keywords:** High Fidelity Simulation Training, Airway Extubation, Patient Safety, Intensive Care Units, Educational Technology.

## Abstract

**Objective:**

To develop and validate the content of an interactive virtual simulation based on a branching scenario for the prevention of unplanned extubation in adult Intensive Care Units.

**Method:**

Technological production developed through the stages of Contextualized Instructional Design and methodological study of content validation. Content validation (objectives, structure and presentation, relevance, general aspects) was carried out by five judges, with the Content Validity Index, Content Validity Ratio, and Content Validity Coefficient being calculated.

**Results:**

The virtual simulation contains five branched scenes, structured and implemented on a free virtual platform. The judges’ average score was 4.3 and the agreement >0.8 in all metrics for validation.

**Conclusion:**

The virtual simulation was developed and validated in terms of content, and is recommended as an educational strategy for nurses. Digital educational technology can support nurses to promote patient safety by preventing the occurrence of unplanned extubation in the intensive care unit.

## INTRODUCTION

Patient safety has been a central concern in healthcare in recent decades, especially the challenges faced by healthcare teams working in complex care units such as Intensive Care Units (ICU). These units treat patients in critical health conditions, which makes them prone to different risks, leading to the occurrence of care-associated adverse events (AE)^(1)^, with emphasis on unplanned extubation (UE).

UE is defined as any type of unintentional and uncontrolled extubation that occurs when the patient himself removes or dislocates the orotracheal tube, or when it is removed by external force applied to the tube, such as during daily care^(2,3)^. It is observed that the occurrence of UE in critically ill patients can result in potential complications, such as injury to the vocal folds and/or trachea, hypoxemia, hemodynamic instability, respiratory failure, brain injury, cardiac arrest, and death^(2,4)^.

The Brazilian Health Regulatory Agency Report describes that 163 UE-related AEs in Brazil were reported from January to December 2021, five of which resulted in deaths^(5)^. In this context, studies point to the need to prevent UE in critically ill patients through continuous assessment of risk factors. Among the risk factors, the control and management of agitation/pain; insufficient sedation and/or weaning from sedation; patient movement and absence of physical restrictions; prone position; assessment of spontaneous breathing; cuff pressure and inadequate fixation of the orotracheal tube; lack of clear weaning and/or extubation procedures; and human resources management^(4,6,7)^ are highlighted.

Given the context presented and the prospect of incorporating new teaching methodologies, which can help promote patient safety and prevent adverse events in the ICU, digital educational technologies (DET) emerge as opportunities for training, updating, or professional qualification. DETs comprise different computerized technologies that provide participants with an active and interactive learning approach, from which it is possible to recreate real-life situations, enabling study within the scope of safe practices and promoting clinical reasoning^(8, 9, 10)^.

In this scenario of transformations, the virtual simulation modality stands out in the health area, defined by the recreation of real-life environments and processes from inputs and outputs performed by a computer, generally associated with a monitor, keyboard or other auxiliary devices, depending on the type of simulation used^(1)^. As a technology capable of contributing to and stimulating problem-solving in an agile way, virtual simulation allows participants to play a central role in exercising different skills^(8, 11, 12, 13, 14)^.

Studies point to the use of virtual simulation as something new in the nursing field, which can contribute to and stimulate the resolution of problems in an agile manner, in addition to simulating the execution of care, as many times as necessary, made possible by the use of a virtual environment^(8,9, 10, 11, 12, 13)^. Based on the possibility of expanding access to a greater number of participants, without time or location restrictions, educational institutions have been introducing virtual simulations as a regular teaching method. The method makes training of technical and behavioral skills possible, allowing participants to experience situations that realistically reproduce everyday life in the health area, from a safe and controlled environment^(8,9,15, 16, 17)^.

Among the different types of virtual simulation, the ones based on branching scenarios stand out. In this method, simulation takes place based on decisions made by the participant, where after watching excerpts of videos, recorded with the participation of actors, the participant answers questions and possible response options from which there may be consequences^(15)^.

During branching scenario simulations, participants have the opportunity to solve problems, learn from mistakes, and receive instant feedback. Evidence in the literature points to effective results from the application of this type of simulation in teaching and learning in nursing, providing a safe and engaging learning environment for the practice of different skills, preparing them for the application of this knowledge in real clinical practice^(16,18,19)^.

Considering the guidelines of the *The International Nursing Association for Clinical Simulation and Learning* (INACSL)^(20)^ and adherence to the United Nations Sustainable Development Goals^(21)^, (axes “Health and well-being” and “Quality education”), this study aimed to develop and validate the content of interactive virtual simulation based on a branching scenario for the prevention of unplanned extubation in adult ICU.

## METHOD

### Design of Study

Technological production based on the stages of Contex­tualized Instructional Design (CID)^(22)^ and methodological study of content validation.

### Population, Selection Criteria, and Sample

Nurses who are experts in Intensive Care participated in the initial meeting with the researchers to choose the simulation theme. The population of judges who participated in the content validation consisted of nurses and teachers who met the following criteria: expertise in patient safety and/or intensive care, working in intensive care assistance and/or management, and/or being member of the Patient Safety Center, and/or being a professor in an Undergraduate and/or Graduate Nursing course, working in Santa Catarina.

### Sample Definition

The non-probabilistic convenience sampling of nurses involved in the initial discussion to define the simulation theme included nurses who worked in the ICU of a public hospital in Florianópolis, SC. These nurses did not participate in the simulation validation stage.

Non-probabilistic convenience sampling of judges for content validation was defined using the snowball technique and analysis of the Lattes Curriculum. The recommendation to include five to 10 judges for content validation was adopted^(23)^.

### Study Protocol

The virtual simulation was developed from the steps of Contextualized Instructional Design: analysis, design, development, implementation, evaluation^(22)^. In the Analysis stage, a meeting was held online (Google Meet platform^®^) with nurses who work in the ICU, to suggest topics related to patient safety in intensive care, considering the adverse events that occurred. At the meeting, the occurrence and type of more serious adverse events at the institution, such as pressure injuries, falls, medication errors, and UE were discussed.

Following the discussion, UE was considered a priority topic for the simulation scenario, due to the serious consequences caused to patients. Based on the definition of the simulation theme, a narrative literature review was carried out (PubMed/MEDLINE, Scopus, *Web of Science*, COCHRANE and SciELO) to search for evidence on risk factors and care for preventing UE, content that supported the technological production.

In the steps design and development of the virtual simulation, the interface (design, colors, images) was defined, in addition to the development of the clinical guide, storyboard (initial structuring of the interconnections of the branching scenario)*,* and the audiovisual script for recording scenes, storing scenes in the cloud, and structuring the decision tree of the simulation scenario. The INACSL guidelines for simulation design were considered for the development of clinical guidelines^(20)^, considering essential criteria of structure, process and results of the activity.

In the Implementation stage, the following were used: I) branching scenarios tool*,* available on the H5P platform^®^, free version, used to structure simulation scenarios; II) Youtube^®^, free version, platform used for storage and organization of audiovisuals used in the simulation; III) Canva^®^, free version, used to create patient records and; IV) Shutterstock^®^, free image bank, used to capture the image of the simulation’s opening screen.

### Data Collection

Initial contact with participants was made via email, containing the researchers’ introduction, title, and objectives of the research. After initial acceptance, participants received the link containing the Free and Informed Consent Form.

Data collection for content validation took place from August to September 2023, over 20 days, on the Moodle platform^®^, where they accessed the guidelines for content evaluation, the clinical guide, the virtual simulation in a branching scenario and the electronic questionnaire (Google Forms^®^) content validation.

The questionnaire contained 24 closed questions that addressed: the proposed objectives (eight questions), the structure and presentation of the simulation (eight questions), the relevance (six questions) and the general aspects (two questions), specifically regarding the possibility of using virtual simulation as a continuing education strategy and the potential for safety culture enhancement^(24)^. For each domain evaluated, judges could include additional evaluations, such as criticisms (negative points), suggestions for improving the simulation and/or praise (positive points).

### Data Analysis and Treatment

The results obtained were organized in electronic spreadsheets (Excel Office^®^ 2010) and statistical software (SPSS V26, 2019 and Minitab 21.2, 2022) was used for analysis. The categories of the validation questionnaire applied were analyzed based on responses on a Likert scale (5-Excellent, 4-Very good, 3-Good, 2-Fair, 1-Poor), with average of ≥3 being considered positive results. For the rigorous analysis of the level of agreement, the following were calculated: the Content Validity Index (CVI) using the modified Kappa^(25)^, to assess inter-judge agreement of the items and the instrument in general, and also, the Content Validity Ratio (CVR), used to assess agreement regarding the relevance of the items and the Content Validity Coefficient (CVC) for construct analysis^(26)^.

The final results of the content validation were classified as: 0.41 to 0.60 – low agreement; 0.61 to 0.80 – moderate agreement; 0.81 to 1 – high agreement, with results equal to or greater than 0.80 being considered positive^(27)^. Cronbach’s alpha coefficient was also calculated to estimate the reliability (internal consistency) of the questionnaire applied in data collection.

### Ethical Aspects

The research contemplates the ethical aspects of Resolution No. 466/2012 of the National Health Council and was approved by the Research Ethics Committee of the Universidade Federal de Santa Catarina, with Opinion No. 5.984.949. All participants were informed about the study and agreed to the Free and Informed Consent Form, with anonymity guaranteed.

## RESULTS

### Development of Interactive Virtual Simulation

After meeting with nurses working in the ICU and searching for scientific evidence on the subject in the literature, a clinical guide was developed, which considered INACSL’s guidelines for planning the design of clinical simulations^(20)^.

In this clinical guide, the theme of the scenario, target audience, prior knowledge of the participant, theoretical basis, learning objectives, duration of the scenario, fidelity of the scenario, simulation modality, human resources, material resources and equipment, moulage, description of the environment, patient’s clinical case/situation, information for participants, script for the simulated patient’s performance, briefing, scenario development, debriefing, and evaluation of participants were described.

**Figure 1:**
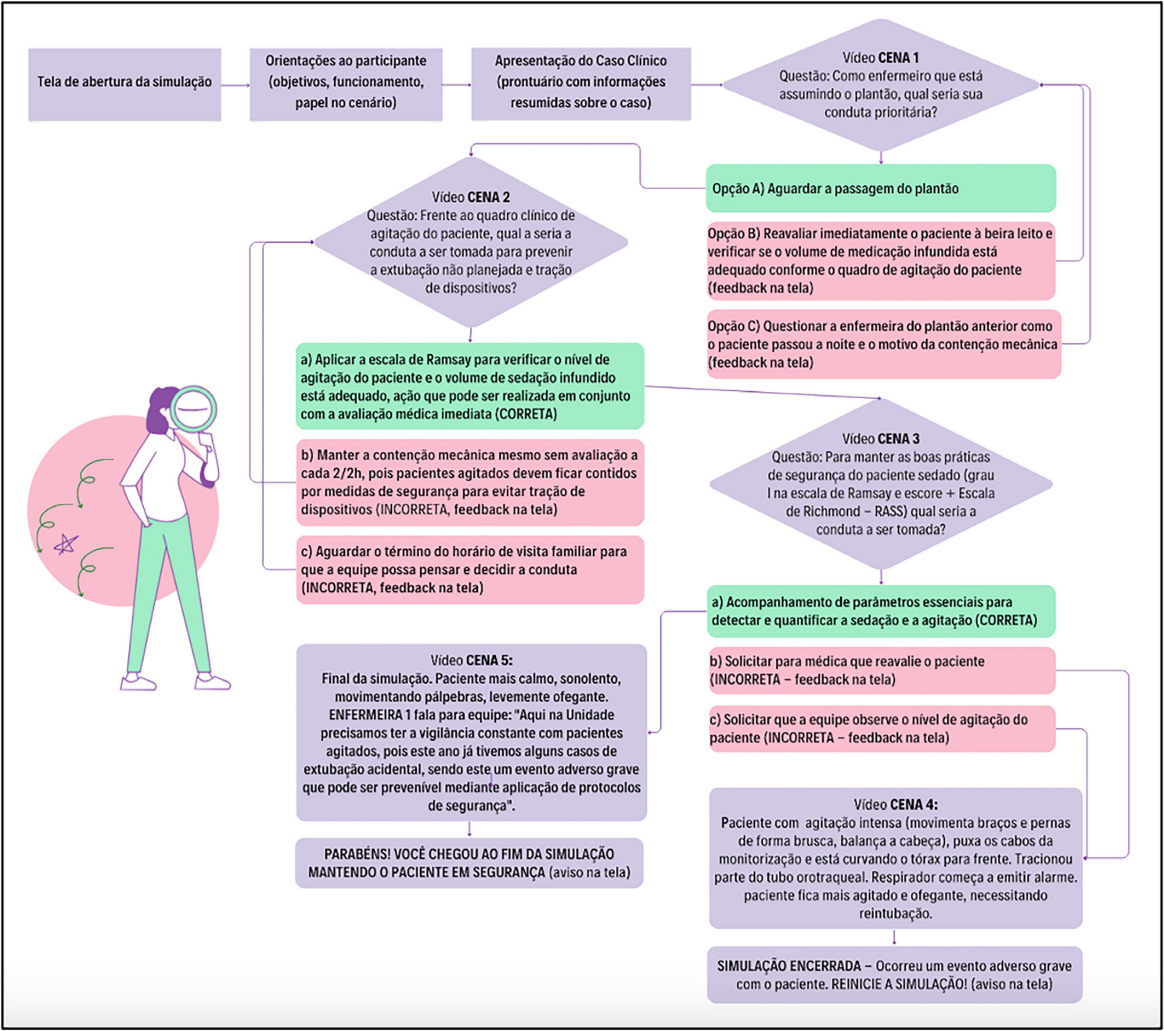
Initial structuring of the decision tree of the simulation scenario – Florianópolis, SC, Brazil, 2023.

An initial structuring of the decision tree of the virtual simulation scenario (Figure 1) was developed from the clinical guide, establishing the basis for the development of the audiovisual script and final structuring of the simulation, later developed on the H5P platform.

Based on the clinical guide and initial scheme of the simulation structure, an audiovisual script was developed by the researchers and formatted in screen writing software (Celtx^®^) by a scholarship holder of the Undergraduate Course of Cinema, for subsequent audiovisual recording of the virtual simulation scenes. The script was registered at the Brazilian National Library, registration certificate no. 883,320, book 1,722, page 37.

The preparation of the audiovisual script of the movement on stage and the speeches of the actors involved in the simulation considered that the information should be passed on in a clear, objective way, using the technical-scientific language of the health area.

The recording location for the simulation scenes was the Simulated Practices Laboratory of the Undergraduate Nursing Course at a public university in southern Brazil, in May 2023. The recording scenario was characterized as an ICU bed, including infusion pumps, multiparameter vital sign monitoring display, mechanical ventilator, hospital bed, in addition to the materials and invasive devices used to characterize the simulated patient.

For the recording, a production team was assembled, consisting of a director, production and audio assistants, and five actors, who played the following roles: Nursing technician; Physician on duty; Patient; Nurse 1 (who was leaving the shift); and Nurse 2 (role assumed by the participant in the simulation). After recording, the five scenes were edited and stored on the Youtube^®^ platform.

The Moodle interface and the virtual simulation developed considered the visual identity foreseen in the macroproject to which this study is linked, applying the sans-serif font (Arial) to the texts, and green color (code 2EB8AD) and the logo of the research macroproject on the Moodle platform.

**Figure 2:**
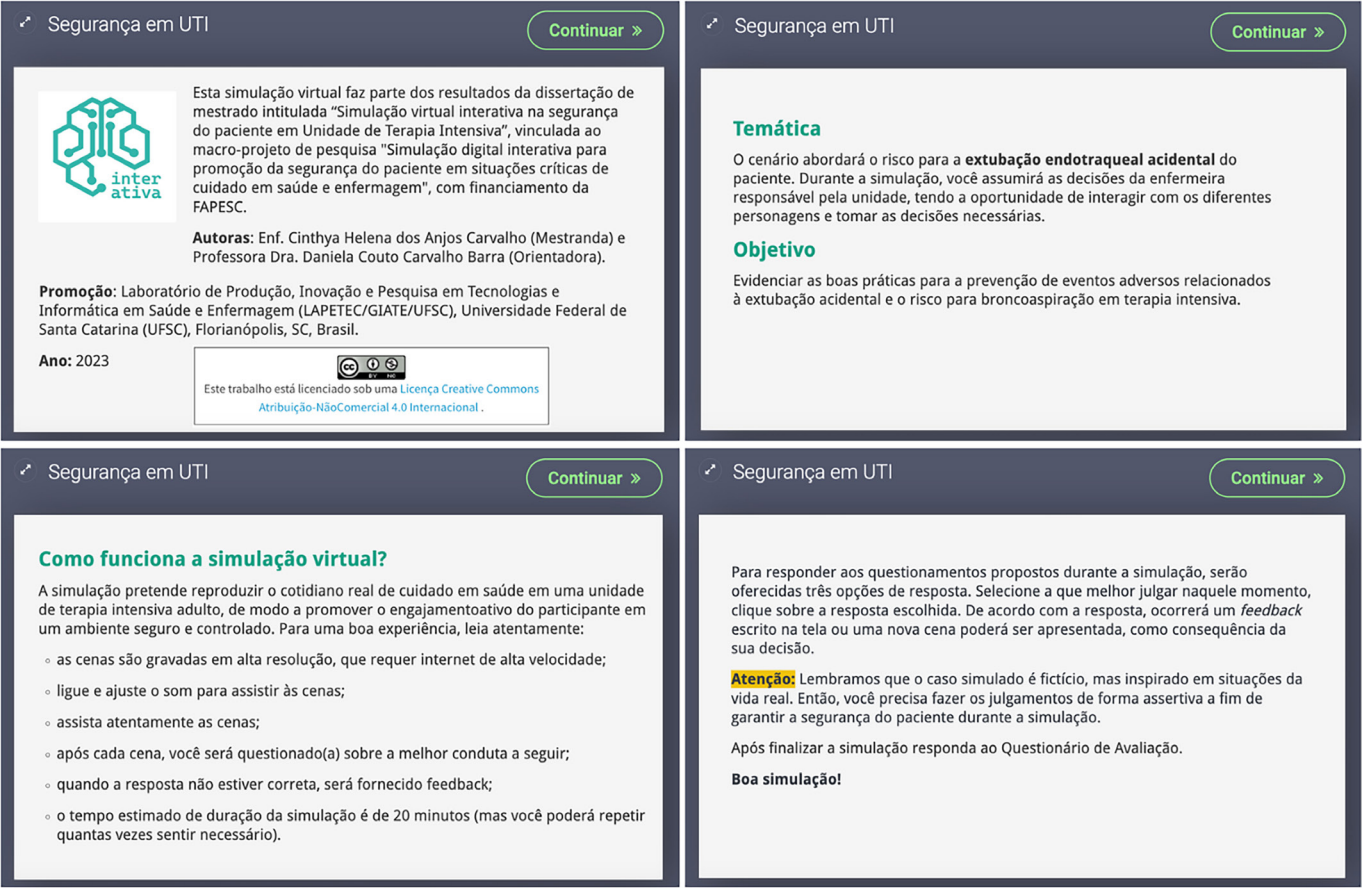
Initial guidance screens for virtual simulation – Florianópolis, SC, Brazil, 2023.

To access the simulation, expert judges were registered on the Moodle Groups platform^®^. The simulation’s initial screens provide instructions for evaluating the simulation, access to the simulation itself in the Virtual Laboratory, and information about the project. Below, the simulation theme, objectives, and important guidelines for a good simulation experience using electronic devices are presented. The guidelines conclude by presenting how to respond to the questions proposed throughout the simulation. Figure 2 shows some of the screens containing essential information for participation in the virtual simulation.

At the beginning of the virtual simulation, the clinical case is presented to the participants, in the format of the patient’s electronic medical record, and from this point onwards the simulation scenes begin to be presented. At the end of each scene, a question was made available with three answer options, with one being correct and two incorrect.

**Figure 3:**
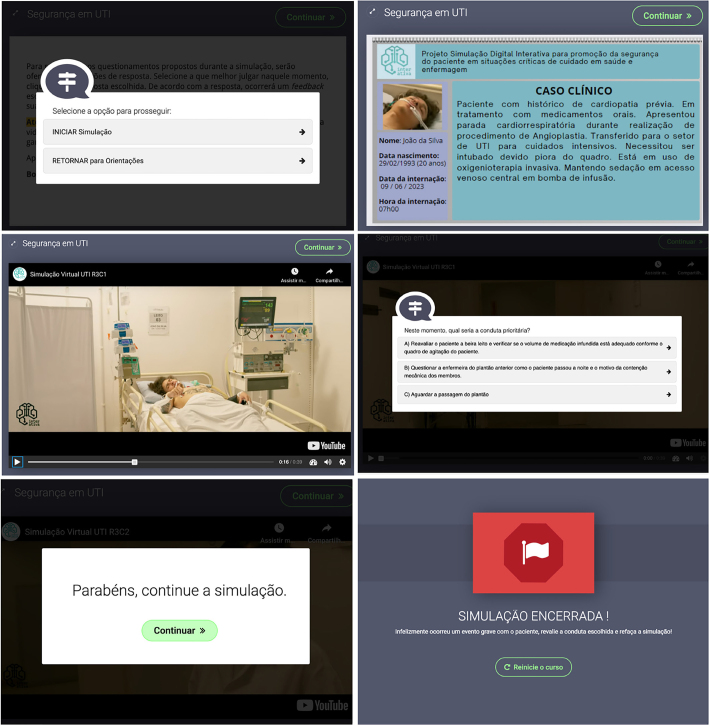
Virtual simulation screens for prevention of UE in ICU – Florianópolis, SC, Brazil, 2023.

For incorrect answers, written feedbacks are presented, based on the scientific literature in the area, and for correct answers the participant is congratulated for the correct answer, and the reference used confirming the scientific evidence is presented. It should be noted that, if the participant clicks on an option that leads to an adverse event, the simulation is terminated, with an informative message appearing on the screen offering the possibility of starting the simulation again. Figure 3 shows some screens from the virtual simulation.

### Validation of Virtual Simulation Based on Branching Scenarios

Ten expert judges (nurses) were invited to perform the evaluation. Five responded to the data collection form, two professors from the ICU area, two from the emergency area, and one from the health management and technology area, all with experience in the area of patient safety, three masters, one specialist, and one doctor. All judges are women, with an average age of 37.2 years and an average training time of 11 years.

When asked about the use of in-person and virtual clinical simulations in their professional practice, the majority reported having already used them, with 100% being in-person simulations and 80% virtual simulations.

The analysis of the Internal Consistency of the questionnaire applied (Cronbach’s alpha) resulted in 0.899.

The results of the content assessment are presented based on the means and standard deviation, and also regarding the CVI, CVC, and CVR, considering the domains Objectives, Structure and presentation, Relevance, and General aspects, in Table 1.

**Table 1:** Results of the evaluation of the interactive virtual simulation according to expert judges (n = 5) – Florianópolis, SC, Brazil, 2023.

Domains/Questions		CVI	CVC	CVR	Mean	SD
**1. Objectives:**	1.1 The contents are consistent with the objective of the clinical simulation scenario	1	0.92	1	4.6	0.89
	1.2 Learning objectives are clear and concise	1	0.84	1	4.2	0.84
	1.3 Scenario content facilitates critical thinking	1	0.84	1	4.2	0.84
	1.4 The information presented is scientifically correct	1	0.88	1	4.4	0.89
	1.5 There is a logical sequence of proposed content	1	0.92	1	4.6	0.55
	1.6 The information presented in the scenario covers the content on prevention of accidental endotracheal extubation in the ICU well.	0.8	0.8	0.6	4	1.22
	1.7 Information/content is important for the quality of care provided	1	0.96	1	4.8	0.45
	1.8 The objective of the scenario invites/instigates changes in participants’ behavior and attitude	0.8	0.76	0.6	3.8	1.30
**2. Structure and presentation**	2.1 The scenario script is appropriate for the participants	1	0.84	1	4.2	0.84
	2.2 The language used is easy to understand by participants	1	0.92	1	4.6	0.55
	2.3 The scenario has an attractive appearance that keeps the participant’s attention	1	0.84	1	4.2	0.84
	2.4 The data is presented in a structured and objective manner	1	0.88	1	4.4	0.55
	2.5 The way the scenario is presented contributes to the participants’ learning	1	0.84	1	4.2	0.84
	2.6 Contextual details provide clues based on desired outcomes	0.8	0.76	0.6	3.8	1.30
	2.7 The patient profile provides sufficient data to make a clinical judgment	1	0.92	1	4.6	0.55
	2.8 The visual composition of the virtual simulations structured on the online platform is attractive and seems organized	0.8	0.72	0.6	3.6	1.14
**3. Relevance**	3.1 The scenario allows knowledge transfer regarding the prevention of accidental endotracheal extubation	1	0.80	1	4	0.71
	3.2 The topic shows key aspects that must be reinforced.	1	0.92	1	4.6	0.55
	3.3 The model allows the transfer and generalization of learning to different contexts	1	0.84	1	4.2	0.84
	3.4 The simulation scenario script proposes the construction of knowledge	1	0.80	1	4	0.71
	3.5 Can be used by healthcare professionals/or educators	0.80	0.84	0.6	4.2	1.30
	3.6 The simulation scenario on prevention of accidental endotracheal extubation in the ICU is of sufficient quality to circulate in scientific circles	1	0.84	1	4.2	0.84
**4. General aspects**	4.1 Simulation can be used as a continuing education strategy to raise awareness about patient safety culture among nurses.	1	0.88	1	4.4	0.89
	4.2 The developed interactive virtual simulation has the potential to promote the strengthening of patient safety culture in care scenarios	1	0.96	1	4.8	0.45
Domain 1		0.95	0.86	0.90	4.3	
Domain 2		0.95	0.84	0.90	4.2	
Domain 3		0.97	0.84	0.93	4.2	
Domain 4		1	0.92	1	4.6	
**Averages**		**0.96**	**0.85**	**0.92**	**4.3**	

Legend: CVI – Content Validity Index; CVC – Content Validity Coefficient; CVR – Content Validity Ratio; SD – standard deviation.

The analysis of agreement among judges (*Fleiss Kappa*) resulted in -0.117 (p 0.010). The results demonstrated a CVI, CVC and CVR >0.80 for the interactive simulation evaluation criteria evaluated. These results demonstrate an acceptable content validity index.

Regarding the assessment of the item “Objectives”, the results can be complemented with the report: *The scenes (actors, scenary and dialogues) are clear and allow the participant, when interacting with the environment, to learn from mistakes and successes, leading to the achievement of the objectives proposed by the study.* (Judge 4).

Some suggestions were made by experts regarding the “Structure and Presentation” of the virtual simulation, as per the statements: *It needs some adjustments: grammar, cleaner design, text positioning and presentation image quality. (...) hosting at another address* (Judge 4); and *(...) when selecting the correct answer, there should also be Feedback with the message that you selected the correct answer and the justification for the intervention. At the end of the simulation, when everything goes correctly and the participant is congratulated for their choices, I suggest that a positive image appears in green, because the image that appears in red (the same one appearing when it ends with the outcome of accidental extubation) denotes something negative.* (Judge 5).

Regarding “Relevance”, Judge 4 complements the results obtained: *The topic is extremely relevant for patient safety and simulation has a lot to contribute to teaching and learning and to nurses’ development of critical thinking and decision-making* (Judge 4)*.*

Regarding “General aspects”, it was investigated whether the continuing education strategy can help raise awareness about patient safety culture and whether the interactive virtual simulation developed has the potential to promote the strengthening of patient safety culture in critical care nursing scenarios, with this comment from one of the experts: *(...) congratulations on building the simulation, the work has great potential for undergraduate, graduate, and continuing education for health professionals. (...). A very relevant point is the feedback to the “nurse” about what would be the best response given the simulation presented.* (Judge 1).

## DISCUSSION

It is understood that interactive virtual simulations, especially from branching scenarios, allow a realistic view of clinical practice, in a safe and controlled environment. Such simulations can contribute to the promotion of patient safety and the prevention of UE in ICUs. In this study, the branching virtual simulation scenario was developed with the participation of nurses working in the ICU, technology and cinema professionals to make it as close to reality as possible and thus provide participants with a safe and controlled environment for decision-making through clinical reasoning.

Studies indicate the following adverse events of UE: need for orotracheal reintubation and increased time of use of invasive mechanical ventilation; increased length of hospital stay; increased risk of hypoxemia, atelectasis, ventilator-associated pneumonia (VAP), tracheal injury, hemodynamic instability, cardiorespiratory arrest, secondary pneumonia, dyspnea, airway trauma; laryngeal edema, greater difficulty for the team to reintubate the patient; and death^(2,4,6,7)^. In view of this scenario, the digital educational technology developed will be able to provide nurses and undergraduate nursing students with the improvement of different skills, supporting them in making their practice safer by establishing care for the prevention of UE.

This way, this developed technology provides a new solution for teaching and learning, thus strengthening the culture of patient safety, since carrying out clinical simulation can contribute to learning and recognition of patterns and clinical decision-making in the health care environment^(10,11,14,17)^.

Other authors emphasize that virtual simulation based on branching scenarios allows the recreation of the daily reality of health professionals in an immersive way, since the participant assumes the role of the nurse in the simulation, an aspect that can favor learning^(12,15, 16, 17, 18, 19)^. Such simulations allow participants to take the lead in the scenario, exercising decision-making safely in a controlled environment, without exposing real patients, thus preserving their physical integrity, as well as situations related to everyday professional ethics^(13,15, 16, 17, 18)^.

Thus, it is believed that the implementation of this type of virtual simulation has the potential to improve learning outcomes, and consequently to promote patient safety, contributing to changing attitudes and behaviors, based on virtual simulation training. Furthermore, it is worth highlighting the possibility of developing technical and non-technical skills, generally consisting of specific procedures for each specialty, at any level of depth. Non-technical skills involve cognitive and social skills that complement the technique for quality and safe professional practice, which is essential for Nurses in the real critical care scenario^(28)^.

From this perspective, it is understood that the implementation of simulations based on branching scenarios allows participants to interact with different characters from the health team and patients, discuss the situations presented in the cases, make decisions based on clinical reasoning, and consequently learn best practices. However, it is worth highlighting that for this activity to occur effectively, a detailed clinical guide has to be constructed, including the learning objectives and skills to be developed by the participants in the proposed scenarios.

The virtual simulation scenario was developed as an opportunity to provide a safe teaching-learning environment, with lower costs and increased possibilities for participation, through online access at any time or place, from mobile or fixed devices. The construction of clinical simulation scenarios is a careful process that must be developed based on scientific evidence, in addition to being subject to peer review by experts in the area, and pilot testing with the target audience. Therefore, this development and updating task is cyclical and must be accompanied by continuous feedback to the simulation participants ^(15, 16, 17, 18, 19)^.

The interface of the virtual simulation developed received special attention during development, being defined as the means by which the user can communicate with a given computerized system, to carry out a given activity. Thus, it aims to promote friendly interaction between humans and machines, through elements arranged on the screen^(29,30)^.

In this study, the stages of the branching scenarios were described and produced with different professionals to ensure the effectiveness of the educational technology and with the perspective that this technology can support nurses regarding the necessary care in UE.

## CONCLUSION

The use of good practices for the development of the branching virtual simulation scenario brought reflections regarding the care provided to patients in the ICU, linked to patient safety, mainly in the prevention of serious adverse events, such as UE.

The results of the evaluations carried out by the judges validated the content and appearance of the technology, with changes being suggested that will be considered before its full implementation. The limitations of the study include the time factor for completing all planned stages and the small sample size.

The strengths highlighted by the judges who evaluated the technology were the search for new objectives and challenges for learning and the importance of this innovative technology for use in the training and continuing education of nursing professionals, and also for the application in the training of undergraduate nursing students.

As a future study perspective, the integration of Large Scale Language Models (LLMs), such as ChatGPT^®^, will be an opportunity to bring new dimensions to the educational experience of Nurses. These models could be used to automatically generate realistic dialogues between characters in simulation scenarios, providing participants with a more authentic interaction that accurately simulates the nuances of human communication in clinical settings. Additionally, they could serve as virtual assistants during simulations, providing evidence-based clinical guidance and immediate feedback on decisions made by students. This would not only deepen the realism of virtual simulations, but would also enable a scalable and interactive form of training, adapting in real time to participants’ responses and the paths chosen in branching scenarios. The potential for personalizing learning from this type of virtual simulation is considerable, allowing simulations that adapt to the professional’s level of experience, reinforcing knowledge acquisition and the development of critical skills. The use of LLMs, therefore, opens a promising field for enriching health education and expanding the frontiers of interactive clinical training.

## DATA AVAILABILITY

The data are available in the FIGSHARE^®^ repository: https://doi.org/10.6084/m9.figshare.24463573.v1. 
