# Supplementary material for: Validation of virtual simulation content for prevention of unplanned extubation in intensive care
Source: Rev Esc Enferm USP. 2025 Jun 9;59:e20240443. doi: 10.1590/1980-220X-REEUSP-2024-0443en (PMC12151300; doi:10.1590/1980-220X-REEUSP-2024-0443en)
Supplement: Translation

# Translation: Validação de conteúdo de simulação virtual para prevenção da extubação não planejada em terapia intensiva*

**DOI:** 10.1590/1980-220X-REEUSP-2024-0443pt

**Authors:** Cinthya Helena dos Anjos Carvalho, Daniela Couto Carvalho Barra, Ana Graziela Alvarez, Neide da Silva Knihs, Grace Teresinha Marcon Dal Sasso, Ricardo João Cruz-Correia, Pedro Miguel Garcez Sardo

**Affiliations:** 1 Universidade Federal de Santa Catarina, Departamento de Enfermagem, Florianópolis, SC, Brasil.; 2 Universidade do Porto, Faculdade de Medicina, Departamento de Medicina Comunitária, Informação e Ciências da Decisão em Saúde, Centro de Investigação em Tecnologias e Serviços de Saúde, Porto, Portugal.; 3 Universidade de Aveiro, Escola Superior de Saúde, Aveiro, Portugal.

**Keywords:** Treinamento com Simulação de Alta Fidelidade, Extubação, Segurança do Paciente, Unidades de Terapia Intensiva, Tecnologia Educacional.

## Abstract

**Objetivo:**

Desenvolver e validar o conteúdo de simulação virtual interativa baseada em cenário ramificado para prevenção da extubação não planejada em Unidades de Terapia Intensiva adulto.

**Método:**

Produção tecnológica desenvolvida por meio das etapas do Design Instrucional Contextualizado e estudo metodológico de validação de conteúdo. A validação de conteúdo (objetivos, estrutura e apresentação, relevância, aspectos gerais) foi realizada por cinco juízes, sendo calculados Índice de Validade de Conteúdo, Razão de Validade de Conteúdo e Coeficiente de Validade de Conteúdo.

**Resultados:**

A simulação virtual contém cinco cenas ramificadas, estruturada e implementada em plataforma virtual gratuita. A nota média dos juízes foi 4,3 e a concordância >0,8 em todas métricas para validação.

**Conclusão:**

A simulação virtual foi desenvolvida e validada quanto ao conteúdo, sendo recomendada como estratégia educacional para enfermeiros. A tecnologia educacional digital poderá apoiar enfermeiros a promover a segurança de pacientes na prevenção da ocorrência de extubação não planejada em unidade de terapia intensiva.

## INTRODUÇÃO

A segurança do paciente é uma preocupação central na área da saúde nas últimas décadas, especialmente os desafios enfrentados por equipes de saúde que atuam em unidades de cuidados complexos como as Unidades de Terapia Intensiva (UTI). Essas unidades tratam pacientes em estado de saúde crítico, o que os torna propensos a diferentes riscos, que podem levar à ocorrência de eventos adversos (EA) associados ao cuidado^(1)^, com destaque para a extubação não planejada (ENP).

A ENP é conceituada como todo tipo de extubação não intencional e não controlada, que ocorre quando o próprio paciente remove ou desloca o tubo orotraqueal, ou ainda, quando ele é removido por força externa aplicada ao tubo, como por exemplo durante os cuidados diários^(2,3)^. Observa-se que a ocorrência da ENP em pacientes criticamente doentes pode resultar em potenciais complicações, como lesão nas cordas vocais e/ou traqueia, hipoxemia, instabilidade hemodinâmica, insuficiência respiratória, lesão cerebral, parada cardíaca e óbito^(2,4)^.

O Relatório da Agência Nacional de Vigilância Sanitária descreve que 163 EA relacionados à ENP no Brasil foram notificados de janeiro a dezembro de 2021, sendo que cinco destes resultaram em óbitos^(5)^. Neste contexto, estudos apontam para a necessidade da prevenção da ENP em pacientes críticos por meio da avaliação contínua dos fatores de riscos. Entre os fatores de risco, destacam-se o controle e a gestão da agitação/*delirium*/dor; sedação insuficiente e/ou desmame da sedação; movimentação do paciente e ausência de restrições físicas; posição prona; avaliação da respiração espontânea; pressão do *cuff* e fixação do tubo orotraqueal inadequada; ausência de procedimentos claros de desmame e/ou de extubação; e gestão dos recursos humanos^(4,6,7)^.

Diante do contexto apresentado e da perspectiva de incorporar novas metodologias de ensino, as quais podem auxiliar na promoção da segurança do paciente e prevenção de eventos adversos em UTI, as tecnologias educacionais digitais (TED) surgem como oportunidades de formação, atualização ou capacitação profissional. As TED compõem diferentes tecnologias informatizadas que oportunizam aos participantes uma abordagem de aprendizagem ativa e interativa, a partir dos quais é possível recriar situações da vida real, possibilitando o estudo no âmbito das práticas seguras e promoção do raciocínio clínico^(8–10)^.

Neste cenário de transformações, a modalidade de simulação virtual destaca-se na área da saúde, definida pela recriação de ambientes e processos da vida real a partir de entradas e saídas realizadas por computador, geralmente associado a um monitor, teclado ou outros dispositivos auxiliares, conforme o tipo de simulação utilizada^(1)^. Como uma tecnologia capaz de contribuir e estimular a resolução de problemas de forma ágil, a simulação virtual permite que participantes tenham um papel central ao exercer diferentes habilidades^(8,11–14)^.

Estudos apontam o uso da simulação virtual como algo novo na área de enfermagem, que pode contribuir e estimular a resolução de problemas de forma ágil, além de simular a execução de cuidados, quantas vezes forem necessárias, possibilitado pelo uso de um ambiente virtual^(8,10–13)^. A partir da possibilidade de ampliar o acesso a um maior número de participantes, sem restrições de tempo ou local, as instituições de ensino vêm introduzindo as simulações virtuais como um método de ensino regular. O método possibilita o treinamento de habilidades técnicas e comportamentais, oportunizando que o participante vivencie situações que reproduzem de modo realista o cotidiano na área da saúde, a partir de um ambiente seguro e controlado^(8,9,15–17)^.

Entre os diferentes tipos de simulação virtual, destacam-se as simulações virtuais baseadas em cenários ramificados. Neste método, a simulação desenrola-se a partir das decisões tomadas pelo participante, onde após assistir trechos de vídeos, gravados com a participação de atores, o participante responde a questões e possíveis opções de resposta a partir das quais poderá haver consequências^(15)^.

Durante as simulações em cenários ramificados, os participantes têm a oportunidade de resolver problemas, aprender a partir dos erros e receber devolutivas instantâneas. Evidências na literatura apontam resultados efetivos da aplicação deste tipo de simulação no ensino-aprendizagem em enfermagem, proporcionando um ambiente de aprendizagem seguro e envolvente para a prática de diferentes habilidades, preparando-os para a aplicação destes conhecimentos na prática clínica real^(16,18,19)^.

Considerando as diretrizes da *The International Nursing Association for Clinical Simulation and Learning* (INACSL)^(20)^ e a aderência aos Objetivos de Desenvolvimento Sustentável da Organização das Nações Unidas^(21)^, (eixos “Saúde e bem-estar” e “Educação de qualidade”), este estudo objetivou desenvolver e validar o conteúdo de simulação virtual interativa baseada em cenário ramificado para a prevenção da extubação não planejada em UTI adulto.

## MÉTODO

### Desenho do Estudo

Produção tecnológica a partir das etapas do Design Instrucional Contextualizado (DIC)^(22)^ e estudo metodológico de validação de conteúdo.

### População, Critérios de Seleção e Amostra

Enfermeiros experts em Terapia Intensiva participaram da reunião inicial com os pesquisadores para escolha da temática da simulação. A população de juízes que participaram da validação do conteúdo foi composta por enfermeiros e docentes que atenderam aos seguintes critérios: expertise em segurança do paciente e/ou terapia intensiva, que atuam na assistência e/ou gerência em terapia intensiva, e/ou membro do Núcleo de segurança do paciente, e/ou docente em curso de Graduação e/ou Pós-graduação em Enfermagem, que atuem em Santa Catarina.

### Definição da Amostra

A amostragem não probabilística por conveniência de enfermeiros envolvidos na discussão inicial para definição da temática da simulação incluiu enfermeiros que atuavam em UTI de um hospital público de Florianópolis, SC. Estes enfermeiros não participaram da etapa de validação da simulação.

A amostragem não probabilística por conveniência de juízes para validação de conteúdo foi definida mediante a técnica bola de neve e análise de Currículo Lattes. Foi adotada a recomendação da inclusão de cinco a 10 juízes para a validação de conteúdo^(23)^.

### Protocolo do Estudo

A simulação virtual foi desenvolvida a partir das etapas do *Design* Instrucional Contextualizado: análise, *design*, desenvolvimento, implementação, avaliação^(22)^. Na etapa de Análise foi realizada reunião *online* (plataforma Google Meet^®^) com enfermeiros de um hospital que atuam em UTI, para a sugestão de temas relacionados à segurança do paciente em terapia intensiva, considerando os eventos adversos ocorridos. Na reunião, foram discutidos a ocorrência e tipo de eventos adversos de maior gravidade ocorridos na instituição, como lesão por pressão, quedas, erros de medicação e ENP.

Após discussão, a ENP foi considerada tema prioritário para o cenário de simulação, devido às graves consequências ocasionadas em pacientes. A partir da definição do tema da simulação foi realizada uma revisão narrativa de literatura (PubMed/MEDLINE, Scopus, *Web of Science*, COCHRANE e SciELO) para busca de evidências sobre os fatores de risco e os cuidados para prevenção da ENP, conteúdo que embasou a produção tecnológica.

Nas etapas *design* e desenvolvimento da simulação virtual foram definidas a interface (desenho, cores, imagens), além do desenvolvimento da guia clínica, *storyboard* (estruturação inicial das interligações do cenário ramificado) e o roteiro audiovisual para a gravação das cenas, armazenamento de cenas em nuvem e estruturação da árvore decisória do cenário de simulação. Para o desenvolvimento das guias clínicas foram consideradas as diretrizes para o desenho de simulações da INACSL^(20)^, considerando critérios essenciais de estrutura, processo e resultados da atividade.

Na etapa de Implementação foram utilizadas: I) ferramenta cenários ramificados (do inglês, *branching scenarios),* disponível na plataforma H5P^®^, versão gratuita, usada para a estruturação dos cenários de simulação; II) Youtube^®^, versão gratuita, plataforma utilizada para armazenamento e organização dos audiovisuais usados na simulação; III) Canva^®^, versão gratuita, utilizado para criação do prontuário do paciente e; IV) Shutterstock^®^, banco de imagens gratuito, usado para captar imagem da tela de abertura da simulação.

### Coleta de Dados

O contato inicial com os participantes foi realizado por e-mail, contendo a apresentação das pesquisadoras, título e objetivos da pesquisa. Após o aceite inicial, os participantes receberam o *link* contendo o Termo de Consentimento Livre e Esclarecido.

A coleta de dados para validação de conteúdo ocorreu de agosto a setembro de 2023, durante 20 dias, na plataforma Moodle^®^, onde acessaram as orientações para avaliação do conteúdo, a guia clínica, a simulação virtual em cenário ramificado e o questionário eletrônico (Google Forms^®^) de validação de conteúdo.

O questionário continha 24 questões fechadas que abordaram: os objetivos propostos (oito questões), a estrutura e apresentação da simulação (oito questões), a relevância (seis questões) e os aspectos gerais (duas questões), especificamente quanto a possibilidade de uso da simulação virtual como estratégia de educação permanente e o potencial para fortalecimento da cultura de segurança^(24)^. Para cada domínio avaliado os juízes poderiam incluir avaliações adicionais, tais como críticas (pontos negativos), sugestões para melhoria da simulação e/ou elogios (pontos positivos).

### Análise e Tratamento dos Dados

Os resultados obtidos foram organizados em planilhas eletrônicas (Excel Office^®^ 2010) e para a análise foi utilizado *software* estatístico (SPSS V26, 2019 e Minitab 21.2, 2022). As categorias do questionário de validação aplicado foram analisadas a partir de respostas em escala de *Likert* (5-Excelente, 4-Muito bom, 3-Bom, 2-Regular, 1-Ruim), sendo considerados resultados positivos médias ≥3. Para a análise rigorosa do grau de concordância foram calculados: o Índice de Validade do Conteúdo (IVC) por meio do Kappa modificado^(25)^, para avaliar a concordância interjuízes dos itens e do instrumento de forma geral, e ainda, a Razão de Validade de Conteúdo (RVC), utilizada para avaliar a concordância quanto à relevância dos itens e o Coeficiente de Validade de Conteúdo (CVC) para análise do constructo^(26)^.

Os resultados finais da validação de conteúdo foram classificados em: 0,41 a 0,60 – baixa concordância; 0,61 a 0,80 – concordância moderada; 0,81 a 1 – alta concordância, sendo considerados resultados positivos iguais ou maiores que 0,80^(27)^. O coeficiente alfa de *Cronbach* também foi calculado, para estimar a confiabilidade (consistência interna) do questionário aplicado na coleta dos dados.

### Aspectos éticos

A pesquisa contempla os aspectos éticos da Resolução nº 466/2012 do Conselho Nacional de Saúde e foi aprovado pelo Comitê de Ética em Pesquisa da Universidade Federal de Santa Catarina, sob Parecer nº 5.984.949. Todos os participantes foram esclarecidos sobre o estudo e concordaram com Termo de Consentimento Livre e Esclarecido, sendo garantido o anonimato.

## RESULTADOS

### Desenvolvimento da Simulação Virtual Interativa

Após reunião com enfermeiros que atuam em UTI e busca de evidências científicas sobre a temática na literatura, foi desenvolvida uma guia clínica, que levou em consideração as diretrizes para o planejamento do desenho de simulações clínicas propostas pela INACSL^(20)^.

Nesta guia clínica, foram descritos o tema do cenário, público-alvo, conhecimento prévio do participante, fundamentação teórica, objetivos da aprendizagem, tempo de duração do cenário, fidelidade do cenário, modalidade da simulação, recursos humanos, recursos materiais e equipamentos, moulage, descrição do ambiente, caso/situação clínica do paciente, informações aos participantes, roteiro do desempenho do paciente simulado, *briefing*, desenvolvimento do cenário, *debriefing* e avaliação dos participantes.

Uma estruturação inicial da árvore decisória do cenário de simulação virtual (Figura 1) foi desenvolvida a partir da guia clínica, estabelecendo a base para o desenvolvimento do roteiro audiovisual e estruturação final da simulação, posteriormente desenvolvida na plataforma H5P.

**Figura 1:** Estruturação inicial da árvore decisória do cenário de simulação – Florianópolis, SC, Brasil, 2023.

A partir da guia clínica e esquema inicial da estruturação da simulação, um roteiro audiovisual foi desenvolvido pelas pesquisadoras e formatado em *software* de roteirização (Celtx^®^) por bolsista do Curso de Graduação em Cinema, para posterior gravação audiovisual das cenas que compuseram a simulação virtual. O roteiro foi registrado na Biblioteca Nacional, certidão de registro n.º 883.320, livro 1.722, folha 37.

A elaboração do roteiro audiovisual (*script*) da movimentação em cena e das falas dos atores envolvidos na simulação considerou que as informações fossem repassadas de forma clara, objetiva, a partir da linguagem técnica-científica da área da saúde.

A locação das gravações das cenas da simulação ocorreu no Laboratório de Práticas Simuladas do Curso de Graduação em Enfermagem de uma universidade pública do sul do Brasil, em maio de 2023. O cenário de gravação foi caracterizado como um leito de UTI, incluindo bombas de infusão, monitor multiparâmetros de monitorização de sinais vitais, ventilador mecânico, cama hospitalar, além dos materiais e dispositivos invasivos usados para caracterização do paciente simulado.

Para a gravação foi reunida uma equipe de produção composta por diretor, assistentes de produção e áudio, e ainda, cinco atores, que interpretaram os papéis: Técnica em enfermagem; Médica plantonista; Paciente; Enfermeira 1 (que estava saindo do plantão); e Enfermeira 2 (papel assumido pelo participante na simulação). Após as gravações, as cinco cenas foram editadas e armazenadas na plataforma Youtube^®^.

A interface do Moodle e da simulação virtual desenvolvida considerou a identidade visual prevista no macroprojeto ao qual este estudo está vinculado, aplicando-se nos textos a fonte não serifada (Arial), e na plataforma Moodle aplicada cor verde (código 2EB8AD) e logotipo do macroprojeto de pesquisa.

Para acessar a simulação os juízes especialistas foram cadastrados na plataforma Moodle Grupos^®^. As telas iniciais da simulação fornecem instruções para avaliação da simulação, o acesso à simulação propriamente dita no Laboratório Virtual e informações sobre o projeto. A seguir, são apresentadas a temática da simulação, objetivos e orientações importantes para uma boa experiência de simulação a partir de dispositivos eletrônicos. As orientações são encerradas apresentando-se a forma de resposta aos questionamentos propostos ao longo da simulação. A Figura 2 apresenta algumas das telas contendo as informações essenciais para participação na simulação virtual.

**Figura 2:** Telas de orientações iniciais para simulação virtual – Florianópolis, SC, Brasil, 2023.

No início da simulação virtual o caso clínico é apresentado aos participantes, no formato de prontuário eletrônico do paciente, sendo que a partir deste ponto as cenas da simulação começam a ser apresentadas. Ao final de cada cena foi disponibilizada uma questão com três opções de respostas, sendo uma opção correta e duas incorretas.

Para as respostas incorretas são apresentados *feedbacks* escritos, baseados na literatura científica da área, e para as respostas corretas o participante é parabenizado pelo acerto, sendo apresentada a referência utilizada que confirma a evidência científica. Destaca-se que, caso o participante clique em uma opção que leve a um evento adverso, a simulação é encerrada, surgindo a mensagem informativa na tela com oferta de possibilidade de iniciar novamente a simulação. A Figura 3 apresenta algumas telas da simulação virtual.

**Figura 3:** Telas da simulação virtual para prevenção da ENP em UTI – Florianópolis, SC, Brasil, 2023.

### Validação da Simulação Virtual Baseada em Cenários Ramificados

Dez juízes especialistas (enfermeiros) foram convidados para a avaliação. Cinco responderam o formulário de coleta de dados, sendo dois docentes da área de UTI, dois da área de emergência e um da área de gestão e tecnologias em saúde, todos com experiência na área de segurança do paciente, sendo três mestres, um especialista e um doutor. Todos os juízes são mulheres, com idade média de 37,2 anos e média do tempo de formação de 11 anos.

Quando questionados sobre o uso de simulações clínicas presenciais e virtuais em sua prática profissional, a maioria relatou já ter utilizado, sendo 100% simulações presenciais e 80% simulações virtuais.

A análise da Consistência Interna do questionário aplicado (Alfa de *Cronbach*) resultou em 0,899.

Os resultados da avaliação de conteúdo são apresentados a partir das médias e desvio padrão, e ainda quanto ao IVC, CVC e CVR, considerando os domínios Objetivos, Estrutura e apresentação, Relevância, e Aspectos gerais, na Tabela 1.

**Tabela 1:** Resultados da avaliação da simulação virtual interativa segundo os juízes especialistas (n = 5) – Florianópolis, SC, Brasil, 2023.

Domínios/Questões		IVC	CVC	CVR	Média	DP
**1. Objetivos**	1.1 Os conteúdos estão coerentes com o objetivo do cenário de simulação clínica	1	0,92	1	4,6	0,89
	1.2 Objetivos de aprendizagem estão claros e concisos	1	0,84	1	4,2	0,84
	1.3 O conteúdo do cenário facilita o pensamento crítico	1	0,84	1	4,2	0,84
	1.4 As informações apresentadas estão cientificamente corretas	1	0,88	1	4,4	0,89
	1.5 Há uma sequência lógica de conteúdo proposto	1	0,92	1	4,6	0,55
	1.6 As informações apresentadas no cenário abrangem bem o conteúdo sobre prevenção de extubação endotraqueal acidental em UTI	0,8	0,8	0,6	4	1,22
	1.7 As informações/conteúdos são importantes para a qualidade da assistência prestada	1	0,96	1	4,8	0,45
	1.8 O objetivo do cenário convida/instiga mudanças de comportamento e atitude dos participantes	0,8	0,76	0,6	3,8	1,30
**2. Estrutura e apresentação**	2.1 O roteiro do cenário é apropriado aos participantes	1	0,84	1	4,2	0,84
	2.2 A linguagem utilizada é de fácil compreensão pelos participantes	1	0,92	1	4,6	0,55
	2.3 O cenário possui visual atraente que mantém a atenção do participante	1	0,84	1	4,2	0,84
	2.4 Os dados estão apresentados de maneira estruturada e objetiva	1	0,88	1	4,4	0,55
	2.5 A forma de apresentação do cenário contribui para o aprendizado dos participantes	1	0,84	1	4,2	0,84
	2.6 Detalhes contextuais fornecem pistas com base em resultados desejados	0,8	0,76	0,6	3,8	1,30
	2.7 O perfil do paciente fornece dados suficientes para a realização de um julgamento clínico	1	0,92	1	4,6	0,55
	2.8 A composição visual das simulações virtuais estruturadas na plataforma online é atrativa e parece organizada	0,8	0,72	0,6	3,6	1,14
**3. Relevância**	3.1 O cenário permite a transferência de conhecimento em relação à prevenção da extubação endotraqueal acidental	1	0,80	1	4	0,71
	3.2 O tema retrata aspectos-chave que devem ser reforçados	1	0,92	1	4,6	0,55
	3.3 O modelo permite a transferência e generalização do aprendizado a diferentes contextos	1	0,84	1	4,2	0,84
	3.4 O roteiro do cenário de simulação propõe a construção de conhecimento	1	0,80	1	4	0,71
	3.5 Pode ser usado por profissionais de saúde/ou educadores	0,80	0,84	0,6	4,2	1,30
	3.6 O cenário de simulação sobre prevenção de extubação endotraqueal acidental em UTI tem qualidade para circular no meio científico	1	0,84	1	4,2	0,84
**4. Aspectos gerais**	4.1 A simulação poderá ser usada como estratégia de educação permanente na sensibilização sobre a cultura de segurança do paciente entre Enfermeiros	1	0,88	1	4,4	0,89
	4.2 A simulação virtual interativa desenvolvida tem potencial para promover o fortalecimento da cultura de segurança do paciente nos cenários de cuidado	1	0,96	1	4,8	0,45
Domínio 1		0,95	0,86	0,90	4,3	
Domínio 2		0,95	0,84	0,90	4,2	
Domínio 3		0,97	0,84	0,93	4,2	
Domínio 4		1	0,92	1	4,6	
**Médias**		**0,96**	**0,85**	**0,92**	**4,3**	

Legenda: IVC – Índice de Validade de Conteúdo; CVC – Coeficiente de Validade de Conteúdo; CVR – Razão de Validade de Conteúdo; DP – desvio padrão.

A análise de concordância entre juízes (*Fleiss de Kappa*) resultou em –0,117 (p 0,010). Os resultados demonstraram um IVC, CVC e CVR >0,80 para os critérios de avaliação da simulação interativa avaliados. Estes resultados evidenciam índice de validade de conteúdo aceitável.

Quanto à avaliação do item “Objetivos”, os resultados podem ser complementados com o relato: *As cenas (atores, cenário e diálogos) são claras e permitem que o participante, ao interagir com o ambiente, aprenda com os erros e acertos, levando ao alcance dos objetivos propostos pelo estudo* (Juiz 4).

Algumas sugestões foram indicadas pelos especialistas quanto à “Estrutura e Apresentação” da simulação virtual, conforme as falas: *Necessita alguns ajustes: gramática, design mais clean, posicionamentos de textos e qualidade de imagem da apresentação. (...) hospedagem em outro endereço* (Juiz 4); e *(...) ao selecionar a resposta correta, também deveria ter um Feedback com a mensagem de que selecionou a resposta correta e a justificativa da intervenção. Ao final da simulação, quando tudo ocorre de maneira correta e o participante é parabenizado por suas escolhas, sugiro que apareça uma imagem positiva e na cor verde, pois, a imagem que aparece, na cor vermelha (a mesma que aparece quando finaliza com o desfecho da extubação acidental), denota algo negativo.* (Juiz 5).

Em relação à “Relevância”, o Juiz 4 complementa os resultados obtidos: *O tema é de extrema relevância para a segurança do paciente e a simulação tem muito a contribuir para o ensino-aprendizagem e para o desenvolvimento do pensamento crítico e tomada de decisão do enfermeiro* (Juiz 4)*.*

Quanto aos “Aspectos gerais”, foi investigado se a estratégia de educação permanente pode ajudar na sensibilização sobre a cultura de segurança do paciente e se a simulação virtual interativa desenvolvida tem potencial para promover o fortalecimento da cultura de segurança do paciente nos cenários de cuidado crítico em enfermagem, havendo este comentário de um dos especialistas: *(...)parabéns pela construção da simulação, o trabalho tem muito potencial para o ensino de graduação, pós-graduação e educação permanente de profissionais de saúde. (...). Um ponto bastante relevante é o feedback ao “enfermeiro” de qual seria a melhor resposta diante da simulação apresentada.* (Juiz 1).

## DISCUSSÃO

Entende-se que as simulações virtuais interativas, especialmente a partir de cenários ramificados, permitem uma visão realista da prática clínica, em ambiente seguro e controlado. Tais simulações podem contribuir para a promoção da segurança do paciente e a prevenção da ENP nas UTIs. Nesse estudo, o cenário ramificado de simulação virtual foi desenvolvido com a participação de enfermeiros que atuam em UTI, profissionais da tecnologia e cinema no sentido de torná-la o mais próximo possível da realidade e assim, oportunizar aos participantes um ambiente seguro e controlado para tomada de decisões por meio do raciocínio clínico.

Estudos apontam como eventos adversos das ENP: necessidade de reintubação orotraqueal e aumento do tempo de uso da ventilação mecânica invasiva; aumento do tempo de internação; aumento do risco de hipoxemia, atelectasia, pneumonia associada a ventilação mecânica (PAV), lesão em traqueia, instabilidade hemodinâmica, parada cardiorrespiratória, pneumonia secundária, dispneia, trauma de vias áreas; edema laríngeo, maior dificuldade por parte da equipe em reintubar o paciente; e óbito^(2,4,6,7)^. Diante deste cenário, a tecnologia educacional digital desenvolvida poderá proporcionar aos enfermeiros e estudantes de graduação em enfermagem o aprimoramento de diferentes habilidades, apoiando-os no sentido de tornar sua prática mais segura a partir do estabelecimento de cuidados para prevenção da ENP.

Desta forma, essa tecnologia desenvolvida oportuniza uma nova solução para o ensino-aprendizagem, fortalecendo assim a cultura de segurança do paciente, visto que a realização de simulação clínica pode contribuir para a aprendizagem e o reconhecimento dos padrões e a tomada de decisão clínica no ambiente de cuidado em saúde^(10,11,14,17)^.

Outros autores reforçam que a simulação virtual baseada em cenários ramificados permite a recriação da realidade cotidiana dos profissionais de saúde de modo imersivo, visto que o participante assume o papel do enfermeiro na simulação, aspecto que pode favorecer a aprendizagem^(12,15–19)^. Tais simulações permitem que os participantes assumam o protagonismo no cenário, exercendo a tomada de decisão de forma segura em um ambiente controlado, sem exposição dos pacientes reais, preservando assim sua integridade física, e ainda as situações relacionadas a ética profissional do cotidiano^(13,15–18)^.

Assim, acredita-se que a implementação deste tipo de simulação virtual tem potencial para melhorar os resultados na aprendizagem, e consequentemente para promoção da segurança do paciente, colaborando para mudança de atitudes e comportamentos, a partir do treinamento por simulação virtual. Ainda, destaca-se a possibilidade de desenvolvimento de habilidades técnicas e não técnicas, constituídas em geral de procedimentos específicos de cada especialidade, em qualquer nível de aprofundamento. As habilidades não técnicas envolvem as competências cognitivas e sociais que complementam a técnica para o desempenho da prática do profissional com qualidade e segurança, aspectos essenciais para a atuação de Enfermeiros no cenário de cuidado crítico real^(28)^.

Nesta perspectiva, compreende-se que a implantação de simulações baseadas em cenários ramificados permitem aos participantes a interação com diferentes personagens da equipe de saúde e pacientes, discussão das situações apresentadas nos casos, tomada de decisão a partir do raciocínio clínico e consequentemente a aprendizagem das melhores práticas. Todavia, vale destacar que para que esta atividade ocorra de maneira efetiva é necessário a construção detalhada da guia clínica, onde constarão os objetivos de aprendizagem e habilidades a serem desenvolvidas pelos participantes nos cenários propostos.

O cenário da simulação virtual foi desenvolvido como uma oportunidade de proporcionar um ambiente de ensino-aprendizagem seguro, com menor custo e ampliação da possibilidade de participação, por meio do acesso online em qualquer hora ou local, a partir de dispositivos móveis ou fixos. A construção dos cenários de simulação clínica é um processo cuidadoso que deve ser desenvolvido com base em evidências científicas, além de ser submetido à revisão por pares com expertise clínica na área e aplicação de teste piloto junto ao público alvo. Logo, essa tarefa de desenvolvimento e atualização é cíclica, devendo ser acompanhada de feedbacks contínuos aos participantes da simulação^(15–19)^.

A interface da simulação virtual desenvolvida recebeu atenção especial durante o desenvolvimento, sendo definida como o meio pelo qual o usuário pode se comunicar com determinado sistema informatizado, para cumprir determinada atividade. Assim, tem por objetivo promover a interação amigável entre seres humanos e máquinas, por meio de elementos dispostos na tela^(29,30)^.

Neste estudo, as etapas dos cenários ramificados foram descritas e produzidas com diferentes profissionais no sentido de assegurar a efetividade da tecnologia educacional e na perspectiva de que esta tecnologia possa apoiar enfermeiros quanto aos cuidados necessários em ENP.

## CONCLUSÃO

O uso das boas práticas para o desenvolvimento do cenário ramificado de simulação virtual trouxe reflexões no que se refere aos cuidados prestados aos pacientes em UTI, interligada à segurança do paciente principalmente na prevenção de eventos adversos graves, como a ENP.

Os resultados das avaliações realizadas pelos juízes validaram conteúdo e aparência da tecnologia, sendo sugeridas alterações que serão consideradas antes da sua total implementação. Como limitações do estudo, considera-se o fator tempo para conclusão de todas as etapas previstas e o pequeno número amostral.

Os pontos fortes evidenciados pelos juízes que avaliaram a tecnologia foram a busca de novos objetivos e desafios para o aprendizado e a importância dessa tecnologia inovadora para o uso na formação e educação permanente dos profissionais Enfermeiros, podendo também ser aplicada na formação de estudantes de graduação em enfermagem.

Como uma perspectiva de estudo futuro, a integração de Modelos de Linguagem de Grande Escala (LLMs), como o ChatGPT^®^, será uma oportunidade de trazer novas dimensões à experiência educacional de Enfermeiros. Estes modelos poderiam ser utilizados para gerar automaticamente diálogos realistas entre personagens nos cenários de simulação, proporcionando aos participantes uma interação mais autêntica que simula com precisão as nuances da comunicação humana em contextos clínicos. Além disso, poderiam servir como assistentes virtuais durante as simulações, oferecendo orientação clínica baseada em evidências e feedback imediato sobre as decisões tomadas pelos estudantes. Isso não apenas aprofundaria o realismo das simulações virtuais, mas também permitiria uma forma escalável e interativa de treinamento, adaptando-se em tempo real às respostas dos participantes e aos caminhos escolhidos nos cenários ramificados. O potencial para personalização do aprendizado a partir deste tipo de simulação virtual é considerável, permitindo simulações que se adaptam ao nível de experiência do profissional, reforçando a aquisição de conhecimento e o desenvolvimento de habilidades críticas. A utilização de LLMs, portanto, abre um campo promissor para o enriquecimento da educação em saúde e para a ampliação das fronteiras do treinamento clínico interativo.

## DISPONIBILIDADE DE DADOS

Os dados encontram-se disponíveis no repositório FIGSHARE^®^: https://doi.org/10.6084/m9.figshare.24463573.v1.

# Translation: Validación del contenido de una simulación virtual para la prevención de la extubación no planificada en cuidados intensivos*

**DOI:** 10.1590/1980-220X-REEUSP-2024-0443es

**Authors:** Cinthya Helena dos Anjos Carvalho, Daniela Couto Carvalho Barra, Ana Graziela Alvarez, Neide da Silva Knihs, Grace Teresinha Marcon Dal Sasso, Ricardo João Cruz-Correia, Pedro Miguel Garcez Sardo

**Affiliations:** 1 Universidade Federal de Santa Catarina, Departamento de Enfermagem, Florianópolis, SC, Brasil.; 2 Universidade do Porto, Faculdade de Medicina, Departamento de Medicina Comunitária, Informação e Ciências da Decisão em Saúde, Centro de Investigação em Tecnologias e Serviços de Saúde, Porto, Portugal.; 3 Universidade de Aveiro, Escola Superior de Saúde, Aveiro, Portugal.

**Keywords:** Enseñanza Mediante Simulación de Alta Fidelidad, Extubación Traqueal, Seguridad del Paciente, Unidades de Cuidados Intensivos, Tecnología Educacional.

## Abstract

**Objetivo:**

Desarrollar y validar el contenido de una simulación virtual interactiva basada en un escenario ramificado para la prevención de la extubación no planificada en Unidades de Cuidados Intensivos de adultos.

**Método:**

Producción tecnológica desarrollada utilizando las etapas del Diseño Instruccional Contextualizado y un estudio metodológico de validación de contenidos. La validación del contenido (objetivos, estructura y presentación, relevancia, aspectos generales) fue realizada por cinco jueces y se calcularon el Índice de Validez del Contenido, Ratio de Validez del Contenido y Coeficiente de Validez del Contenido.

**Resultados:**

La simulación virtual contiene cinco escenarios ramificados, estructurados e implementados en una plataforma virtual libre. La puntuación media de los jueces fue de 4,3 y la concordancia >0,8 en todas las métricas de validación.

**Conclusión:**

La simulación virtual fue desarrollada y validada en términos de contenido, y se recomienda como estrategia educativa para enfermeros. La tecnología educativa digital puede apoyar a las enfermeras en la promoción de la seguridad del paciente mediante la prevención de la ocurrencia de la extubación no planificada en la unidad de cuidados intensivos.

## INTRODUCCIÓN

La seguridad del paciente ha sido una preocupación central en salud durante las últimas décadas, especialmente los retos a los que se enfrentan los equipos sanitarios que trabajan en unidades de cuidados complejos como las Unidades de Cuidados Intensivos (UCI). Estas unidades tratan a pacientes en estado crítico, lo que las hace propensas a diferentes riesgos que pueden conducir a la aparición de acontecimientos adversos (AA) asociados a la asistencia^(1)^, especialmente la extubación no planificada (ENP).

La extubación no planificada se define como cualquier tipo de extubación no intencionada y no controlada que se produce cuando el paciente retira o desplaza el tubo orotraqueal, o cuando éste se retira por una fuerza externa aplicada al tubo, como ocurre durante los cuidados diarios^(2,3)^. Se ha observado que la aparición de ENP en pacientes críticos puede dar lugar a complicaciones potenciales como lesiones en las cuerdas vocales y/o la tráquea, hipoxemia, inestabilidad hemodinámica, insuficiencia respiratoria, daño cerebral, parada cardiaca y muerte^(2,4)^.

El Informe de la Agencia Nacional de Vigilancia Sanitaria describe que de enero a diciembre de 2021 se notificaron 163 EA relacionados con la ENP en Brasil, cinco de los cuales resultaron en muertes^(5)^. En este contexto, los estudios apuntan a la necesidad de prevenir la ENP en pacientes críticos mediante la evaluación continua de los factores de riesgo. Los factores de riesgo incluyen el control y manejo de la *agitación/delirio/dolor*; sedación insuficiente y/o destete de la sedación; movimiento del paciente y ausencia de restricciones físicas; posición prona; evaluación de la respiración espontánea; presión *del manguito* y fijación inadecuada del tubo orotraqueal; ausencia de procedimientos claros de destete y/o extubación; y gestión de recursos humanos^(4,6,7)^.

Ante este contexto y la perspectiva de incorporar nuevas metodologías docentes que ayuden a promover la seguridad del paciente y prevenir eventos adversos en la UCI, las tecnologías educativas digitales (TED) han surgido como oportunidades de formación, actualización o cualificación profesional. Las TEDs comprenden diferentes tecnologías informatizadas que proporcionan a los participantes un enfoque de aprendizaje activo e interactivo, a partir del cual es posible recrear situaciones de la vida real, posibilitando el estudio en el contexto de prácticas seguras y la promoción del razonamiento clínico^(8–10)^.

En este escenario de transformaciones, se destaca en el área de la salud la modalidad de simulación virtual, definida como la recreación de ambientes y procesos de la vida real a partir de entradas y salidas realizadas por una computadora, generalmente asociada a un monitor, teclado u otros dispositivos auxiliares, dependiendo del tipo de simulación utilizada^(1)^. Como tecnología capaz de contribuir y estimular la resolución ágil de problemas, la simulación virtual permite a los participantes desempeñar un papel central en el ejercicio de diferentes habilidades^(8, 11–14)^.

Los estudios señalan el uso de la simulación virtual como algo novedoso en el campo de la enfermería, que puede contribuir y estimular la resolución ágil de problemas, así como simular la ejecución de cuidados tantas veces como sea necesario, posibilitado por el uso de un entorno virtual^(8,10–13)^. Con la posibilidad de ampliar el acceso a un mayor número de participantes, sin restricciones de tiempo o localización, las instituciones de enseñanza vienen introduciendo las simulaciones virtuales como método habitual de enseñanza. El método permite el entrenamiento de habilidades técnicas y de comportamiento, dando a los participantes la oportunidad de experimentar situaciones que reproducen de forma realista la vida cotidiana en el sector sanitario, desde un entorno seguro y controlado^(8,9,15–17)^.

Entre los distintos tipos de simulación virtual, destacan las simulaciones virtuales basadas en escenarios ramificados. En este método, la simulación se desarrolla a partir de decisiones tomadas por el participante, en las que, tras ver videoclips grabados con la participación de actores, el participante responde a preguntas y posibles opciones de respuesta de las que pueden derivarse consecuencias^(15)^.

Durante las simulaciones en escenarios ramificados, los participantes tienen la oportunidad de resolver problemas, aprender de los errores y recibir retroalimentación instantánea. Las evidencias en la literatura apuntan a los resultados efectivos de aplicar este tipo de simulación a la enseñanza y aprendizaje en enfermería, proporcionando un entorno de aprendizaje seguro y atractivo para practicar diferentes habilidades, preparándoles para aplicar estos conocimientos en la práctica clínica real^(16,18,19)^.

Teniendo en cuenta las directrices de *The International Nursing Association for Clinical Simulation and Learning* (INACSL)^(20)^ y la adhesión a los Objetivos de Desarrollo Sostenible de las Naciones Unidas^(21)^, (ejes «Salud y bienestar» y «Educación de calidad»), este estudio tuvo como objetivo desarrollar y validar el contenido de una simulación virtual interactiva basada en un escenario ramificado para la prevención de la extubación no planificada en una UCI de adultos.

## MÉTODO

### Diseño del Estudio

Producción tecnológica basada en las etapas del Diseño Instruccional Contextualizado (DIC)^(22)^ y un estudio metodológico de validación de contenidos.

### Población, Criterios de Selección y Muestra

Las enfermeras especializadas en cuidados intensivos participaron en la reunión inicial con los investigadores para elegir el tema de la simulación. La población de jueces que participó de la validación de contenido fue constituida por enfermeros y profesores que atendieran a los siguientes criterios: especialización en seguridad del paciente y/o cuidados intensivos, actuación en cuidados intensivos y/o gestión, y/o miembro del Centro de Seguridad del Paciente, y/o profesor en curso de pregrado y/o posgrado en enfermería, actuación en Santa Catarina.

### Definición de la Muestra

La muestra de conveniencia no probabilística de enfermeros que participaron en la discusión inicial para definir el tema de la simulación incluyó enfermeros que trabajaban en la UCI de un hospital público de Florianópolis, SC. Estas enfermeras no participaron en la etapa de validación de la simulación.

La muestra de conveniencia no probabilística de jueces para la validación del contenido se definió utilizando la técnica de bola de nieve y el análisis de currículo vitae de la plataforma Lattes de Brasil. Se adoptó la recomendación de incluir de cinco a diez jueces para la validación del contenido^(23)^.

### Protocolo del Estudio

La simulación virtual se desarrolló utilizando las etapas del *Diseño* Instruccional Contextualizado: análisis, *diseño*, desarrollo, implementación y evaluación^(22)^. En la etapa de Análisis, se realizó una reunión *online* (plataforma Google Meet^®^) con enfermeros de un hospital que trabajan en la UCI, para sugerir temas relacionados con la seguridad del paciente en cuidados intensivos, considerando los eventos adversos ocurridos. En la reunión se discutió la ocurrencia y el tipo de los principales eventos adversos en la institución, tales como lesiones por presión, caídas, errores de medicación y ENP.

Tras el debate, se consideró que la ENP era un tema prioritario para el escenario de la simulación, debido a las graves consecuencias causadas a los pacientes. Una vez definido el tema de la simulación, se realizó una revisión narrativa de la literatura (PubMed/MEDLINE, Scopus, *Web of Science*, COCHRANE y SciELO) para buscar evidencias sobre los factores de riesgo y las precauciones para prevenir la ENP, contenidos que sirvieron de base para la producción tecnológica.

En las etapas de *diseño* y desarrollo de la simulación virtual se definió la interfaz (diseño, colores, imágenes), el desarrollo de la guía clínica, el *storyboard* (estructuración inicial de las interconexiones del escenario ramificado) y el guión audiovisual para la grabación de las escenas, el almacenamiento de las escenas en la nube y la estructuración del árbol de decisiones del escenario de simulación. Para el desarrollo de las guías clínicas, se tuvieron en cuenta las directrices del INACSL^(20)^ para el diseño de simulaciones, considerando criterios esenciales de estructura, proceso y resultados de la actividad.

En la etapa de Implementación, se utilizaron las siguientes herramientas: I) herramienta de ramificación de escenarios (branching scenarios), disponible en la plataforma H5P^®^, versión gratuita, utilizada para estructurar los escenarios de simulación; II) YouTube^®^, versión gratuita, plataforma utilizada para almacenar y organizar los audiovisuales utilizados en la simulación; III) Canva^®^, versión gratuita, utilizada para crear la historia clínica del paciente y; IV) Shutterstock^®^, banco de imágenes gratuito, utilizado para capturar la imagen de la pantalla de apertura de la simulación.

### Recolección de Datos

El contacto inicial con los participantes se realizó por correo electrónico, con una presentación de los investigadores, el título y los objetivos de la investigación. Tras la aceptación inicial, los participantes recibieron un *enlace* con el formulario de consentimiento informado.

La recolección de datos para la validación de contenidos se realizó de agosto a septiembre de 2023, durante 20 días, en la plataforma Moodle^®^, donde accedieron a las guías de evaluación de contenidos, la guía clínica, la simulación virtual en un escenario ramificado y el cuestionario electrónico (Google Forms^®^) para la validación de contenidos.

El cuestionario contenía 24 preguntas cerradas que abordaban: los objetivos propuestos (ocho preguntas), la estructura y presentación de la simulación (ocho preguntas), la relevancia (seis preguntas) y aspectos generales (dos preguntas), específicamente en relación con la posibilidad de utilizar la simulación virtual como estrategia de formación continuada y el potencial para fortalecer la cultura de seguridad^(24)^. Para cada ámbito evaluado, los jueces podían incluir evaluaciones adicionales, como críticas (puntos negativos), sugerencias para mejorar la simulación y/o elogios (puntos positivos).

### Análisis y Tratamiento de Los Datos

Los resultados obtenidos se organizaron en hojas de cálculo (Excel Office^®^ 2010) y se utilizó *software* estadístico para su análisis (SPSS V26, 2019 y Minitab 21.2, 2022). Las categorías del cuestionario de validación aplicado se analizaron mediante una escala *de Likert* (5-Excelente, 4-Muy bueno, 3-Bueno, 2-Regular, 1-Malo), considerándose los resultados positivos puntuaciones medias 33. Para analizar rigurosamente el grado de acuerdo, se calcularon: el Índice de Validez de Contenido (IVC) utilizando el Kappa modificado^(25)^, para evaluar el acuerdo inter-jueces de los ítems y del instrumento en general, y también el Ratio de Validez de Contenido (RVC), utilizado para evaluar el acuerdo sobre la relevancia de los ítems y el Coeficiente de Validez de Contenido (CVC) para analizar el constructo^(26)^.

Los resultados finales de la validación del contenido se clasificaron como: 0,41 a 0,60 – acuerdo bajo; 0,61 a 0,80 – acuerdo moderado; 0,81 a 1 – acuerdo alto, con resultados positivos iguales o superiores a 0,80^(27)^. También se calculó el coeficiente alfa de *Cronbach* para estimar la fiabilidad (consistencia interna) del cuestionario utilizado para recoger los datos.

### Aspectos éticos

El estudio cumple con los aspectos éticos de la Resolución 466/2012 del Consejo Nacional de Salud y fue aprobado por el Comité de Ética en Investigación de la Universidad Federal de Santa Catarina, bajo el Informe Nº 5.984.949. Todos los participantes fueron informados sobre el estudio y aceptaron un Formulario de Consentimiento Libre e Informado, garantizando su anonimato.

## RESULTADOS

### Desarrollo de la Simulación Virtual Interactiva

Tras reunirse con enfermeras de UCI y buscar evidencia científica sobre el tema en la literatura, se elaboró una guía clínica que tuvo en cuenta las directrices para la planificación del diseño de simulaciones clínicas propuestas por el INACSL^(20)^.

Esta guía clínica describe el tema del escenario, el público objetivo, los conocimientos previos de los participantes, la base teórica, los objetivos de aprendizaje, la duración del escenario, la fidelidad del escenario, la modalidad de simulación, los recursos humanos, los recursos materiales y el equipamiento, el moulage, la descripción del entorno, el caso/situación clínica del paciente, la información para los participantes, el guión de actuación del paciente simulado, el *briefing*, el desarrollo del escenario, el *debriefing* y la evaluación de los participantes.

A partir de la guía clínica, se desarrolló una estructuración inicial del árbol de decisión del escenario de simulación virtual (Figura 1), estableciendo la base para el desarrollo del guión audiovisual y la estructuración final de la simulación, posteriormente desarrollada en la plataforma H5P.

**Figura 1:** Estructuración inicial del árbol de decisión del escenario de simulación – Florianópolis, SC, Brasil, 2023.

Con base en la pauta clínica y en la estructura inicial de la simulación, un guión audiovisual fue desarrollado por los investigadores y formateado en un *software de* escritura de guiones Celtx^®^ por un estudiante de pregrado de cine, para la posterior grabación audiovisual de las escenas que componían la simulación virtual. El guión fue registrado en la Biblioteca Nacional, certificado de registro nº 883.320, libro 1.722, folio 37.

El guión audiovisual de las escenas y las líneas pronunciadas por los actores que participaron en la simulación garantizaron que la información se transmitiera de forma clara y objetiva, utilizando el lenguaje técnico-científico del área de la salud.

Las escenas de la simulación fueron grabadas en el Laboratorio de Prácticas Simuladas del Curso de Pregrado en Enfermería de una universidad pública del sur de Brasil, en mayo de 2023. El escenario de grabación fue caracterizado como una cama de UCI, incluyendo bombas de infusión, un monitor multiparamétrico para monitorización de los signos vitales, un ventilador mecánico, una cama de hospital, así como los materiales y dispositivos invasivos utilizados para caracterizar al paciente simulado.

Para la grabación, se formó un equipo de producción compuesto por un director, asistentes de producción y audio, así como cinco actores que interpretaron los papeles: Técnico de enfermería; Médico de guardia; Paciente; Enfermera 1 (que abandonaba la guardia); y Enfermera 2 (el papel asumido por el participante en la simulación). Tras la grabación, se editaron las cinco escenas y se almacenaron en la plataforma Youtube^®^.

La interfaz de Moodle y la simulación virtual desarrollada tuvieron en cuenta la identidad visual del macroproyecto al que está vinculado este estudio, utilizando una fuente sans-serif (Arial) para los textos y un color verde (código 2EB8AD) para la plataforma Moodle y el logotipo del macroproyecto de investigación.

Para acceder a la simulación, los jueces expertos se registraron en la plataforma Moodle Grupos^®^. Las pantallas iniciales de la simulación proporcionan las instrucciones para evaluar la simulación, el acceso a la propia simulación en el Laboratorio Virtual e información sobre el proyecto. A continuación, se presenta el tema de la simulación, los objetivos y las pautas importantes para una buena experiencia de simulación utilizando dispositivos electrónicos. Las directrices terminan con cómo responder a las preguntas planteadas a lo largo de la simulación. La figura 2 muestra algunas de las pantallas que contienen la información esencial para participar en la simulación virtual.

**Figura 2:** Pantallas de orientación inicial para la simulación virtual – Florianópolis, SC, Brasil, 2023.

Al inicio de la simulación virtual, se presenta el caso clínico a los participantes, en el formato de la historia clínica electrónica del paciente, y a partir de ahí se empiezan a presentar las escenas de la simulación. Al final de cada escena, se plantea una pregunta con tres opciones de respuesta, una correcta y dos incorrectas.

Para las respuestas incorrectas, se presenta *un feedback* escrito, basado en la literatura científica del área, y para las respuestas correctas, se felicita al participante por acertar, y se presenta la referencia utilizada que confirma la evidencia científica. Cabe señalar que si el participante hace clic en una opción que conduce a un evento adverso, la simulación se da por terminada y aparece un mensaje informativo en la pantalla que ofrece la posibilidad de iniciar de nuevo la simulación. La figura 3 muestra algunas de las pantallas de la simulación virtual.

**Figura 3:** Pantallas de la simulación virtual para la prevención de ENP en la UCI – Florianópolis, SC, Brasil, 2023.

### Validación de la Simulación Virtual Basada en Escenarios Ramificados

Diez jueces expertos (enfermeros) fueron invitados a la evaluación. Cinco de ellos respondieron al formulario de recogida de datos, de los cuales dos eran docentes en el área de UCI, dos en el área de urgencias y uno en el área de gestión y tecnologías en salud, todos con experiencia en el área de seguridad del paciente, tres de ellos eran máster, uno especialista y otro médico. Todos los jueces eran mujeres, con una edad media de 37,2 años y un periodo medio de formación de 11 años.

Cuando se les preguntó sobre el uso de simulaciones clínicas presenciales y virtuales en su práctica profesional, la mayoría afirmó haberlas utilizado ya, con un 100% de simulaciones presenciales y un 80% de simulaciones virtuales.

El análisis de la Consistencia Interna del cuestionario aplicado (Alfa de *Cronbach*) dio como resultado 0,899.

Los resultados de la evaluación del contenido se presentan en términos de media y desviación estándar, así como de IVC, RVC y CVC, considerando los dominios Objetivos, Estructura y presentación, Relevancia y Aspectos generales, en la Tabla 1.

**Tabla 1:** Resultados de la evaluación de la simulación virtual interactiva según los jueces expertos (n = 5) – Florianópolis, SC, Brasil, 2023.

Dominios/Preguntas		IVC	CVC	RVC	Media	DE
**1. Objetivos**	1.1 Los contenidos son coherentes con el objetivo del escenario de simulación clínica	1	0,92	1	4,6	0,89
	1.2 Los objetivos de aprendizaje son claros y concisos	1	0,84	1	4,2	0,84
	1.3 El contenido del escenario facilita el pensamiento crítico	1	0,84	1	4,2	0,84
	1.4 La información presentada es científicamente correcta	1	0,88	1	4,4	0,89
	1.5 El contenido propuesto sigue una secuencia lógica	1	0,92	1	4,6	0,55
	1.6 La información presentada en el escenario cubre bien el contenido sobre la prevención de la extubación endotraqueal accidental en la UCI.	0,8	0,8	0,6	4	1,22
	1.7 La información/el contenido es importante para la calidad de la atención prestada	1	0,96	1	4,8	0,45
	1.8 El objetivo del escenario invita/instiga cambios en el comportamiento y la actitud de los participantes	0,8	0,76	0,6	3,8	1,30
**2. Estructura y presentación**	2.1 El guión del escenario es adecuado para los participantes	1	0,84	1	4,2	0,84
	2.2 El lenguaje utilizado es fácil de entender para los participante	1	0,92	1	4,6	0,55
	2.3 El escenario tiene un aspecto atractivo que mantiene la atención del participante	1	0,84	1	4,2	0,84
	2.4 Los datos se presentan de forma estructurada y objetiva	1	0,88	1	4,4	0,55
	2.5 La forma en que se presenta el escenario contribuye al aprendizaje de los participantes	1	0,84	1	4,2	0,84
	2.6 Los detalles contextuales proporcionan pistas basadas en los resultados deseados	0,8	0,76	0,6	3,8	1,30
	2.7 El perfil del paciente proporciona datos suficientes para emitir un juicio clínico	1	0,92	1	4,6	0,55
	2.8 La composición visual de las simulaciones virtuales estructuradas en la plataforma en línea es atractiva y parece organizada	0,8	0,72	0,6	3,6	1,14
**3. Relevancia**	3.1 El escenario permite la transferencia de conocimientos relativos a la prevención de la extubación endotraqueal accidental	1	0,80	1	4	0,71
	3.2 El tema retrata aspectos clave que deben reforzarse	1	0,92	1	4,6	0,55
	3.3 El modelo permite transferir y generalizar el aprendizaje a diferentes contextos	1	0,84	1	4,2	0,84
	3.4 El guión del escenario de simulación propone la construcción del conocimiento	1	0,80	1	4	0,71
	3.5 Puede ser utilizado por profesionales sanitarios y/o educadores	0,80	0,84	0,6	4,2	1,30
	3.6 El escenario de simulación sobre la prevención de la extubación endotraqueal accidental en la UCI tiene calidad para circular en la comunidad científica	1	0,84	1	4,2	0,84
**4. Aspectos generales**	4.1 La simulación puede utilizarse como estrategia de educación permanente para concienciar a los enfermeros sobre la cultura de la seguridad del paciente.	1	0,88	1	4,4	0,89
	4.2 La simulación virtual interactiva desarrollada tiene el potencial de promover una cultura de seguridad del paciente en los entornos asistenciales	1	0,96	1	4,8	0,45
Dominio 1		0,95	0,86	0,90	4,3	
Dominio 2		0,95	0,84	0,90	4,2	
Dominio 3		0,97	0,84	0,93	4,2	
Dominio 4		1	0,92	1	4,6	
**Medias**		**0,96**	**0,85**	**0,92**	**4,3**	

Abreviaturas – IVC: índice de validez del contenido; CVC: coeficiente de validez del contenido; RVC: ratio de validez del contenido; DE: desviación estándar.

El análisis de concordancia entre jueces (*Kappa’s Fleiss*) resultó en -0,117 (p 0,010). Los resultados mostraron un CVI, CVC y RVC >0,80 para los criterios de evaluación de la simulación interactiva evaluados. Estos resultados muestran un índice de validez de contenido aceptable.

En cuanto a la evaluación del ítem «Objetivos», los resultados pueden complementarse con el informe: *Las escenas (actores, escenario* y diálogos) *son claras y permiten al participante, al interactuar con el entorno, aprender de los errores y aciertos, lo que conduce al logro de los objetivos propuestos por el estudio* (Juez 4).

Los expertos hicieron algunas sugerencias sobre la «Estructura y presentación» de la simulación virtual, de acuerdo con las siguientes afirmaciones: *Necesita algunos ajustes: gramática, diseño* más *limpio, posicionamiento de los textos y calidad de imagen de la presentación. (...) alojamiento en otra dirección* (Juez 4); y *(...) al seleccionar la respuesta correcta, debería haber también un Feedback con el mensaje de que ha seleccionado la respuesta correcta y la justificación de la intervención. Al final de la simulación, cuando todo va bien y se felicita al participante por sus elecciones, sugiero que aparezca una imagen positiva en verde, porque la imagen que aparece en rojo (la misma que aparece al final con el resultado de la extubación accidental) denota algo negativo* (Juez 5).

En relación a la «Relevancia», el Juez 4 complementa los resultados obtenidos: *El tema es extremadamente relevante para la seguridad del paciente y la simulación tiene mucho que aportar a la enseñanza y el aprendizaje y al desarrollo del pensamiento crítico y la toma de decisiones de las enfermeras* (Juez 4)*.*

En cuanto a los «Aspectos generales», se indagó si la estrategia de educación continua puede ayudar a sensibilizar sobre la cultura de seguridad del paciente y si la simulación virtual interactiva desarrollada tiene potencial para promover el fortalecimiento de la cultura de seguridad del paciente en escenarios críticos de atención de enfermería, comentando uno de los expertos: *(...) felicitaciones por la construcción de la simulación, el trabajo tiene mucho potencial para la docencia de pregrado y posgrado y la educación continua de los profesionales de la salud (...). Un punto muy relevante es la retroalimentación a la «enfermera*» sobre *cuál sería la mejor respuesta a la simulación presentada.* (Juez 1).

## DISCUSIÓN

Se entiende que las simulaciones virtuales interactivas, especialmente las basadas en escenarios ramificados, proporcionan una visión realista de la práctica clínica en un entorno seguro y controlado. Dichas simulaciones pueden contribuir a promover la seguridad del paciente y a prevenir las ENP en las UCI. En este estudio, el escenario de simulación virtual ramificado se desarrolló con la participación de profesionales de enfermería, tecnología y cinematografía de las UCI para que fuera lo más parecido posible a la realidad y proporcionar así a los participantes un entorno seguro y controlado para la toma de decisiones mediante el razonamiento clínico.

Los estudios señalan como acontecimientos adversos en la ENP los siguientes necesidad de reintubación orotraqueal y aumento del tiempo de uso de ventilación mecánica invasiva; aumento de la duración de la estancia; aumento del riesgo de hipoxemia, atelectasia, neumonía asociada al ventilador (NAV), lesión traqueal, inestabilidad hemodinámica, parada cardiorrespiratoria, neumonía secundaria, disnea, traumatismo de la vía aérea; edema laríngeo, mayor dificultad por parte del equipo para re-intubar al paciente; y muerte^(2,4,6,7)^. Ante este escenario, la tecnología educativa digital desarrollada podría proporcionar a las enfermeras y estudiantes de enfermería de pregrado diferentes habilidades, apoyándoles para que su práctica sea más segura, estableciendo cuidados para la prevención de la ENP.

De esta forma, la tecnología desarrollada aporta una nueva solución para la enseñanza y el aprendizaje, fortaleciendo así la cultura de seguridad del paciente, ya que la simulación clínica puede contribuir al aprendizaje y reconocimiento de patrones y toma de decisiones clínicas en el ámbito sanitario^(10,11,14,17)^.

Otros autores refuerzan que la simulación virtual basada en escenarios ramificados permite recrear la realidad cotidiana de los profesionales sanitarios de forma inmersiva, ya que el participante asume el papel de la enfermera en la simulación, aspecto que puede favorecer el aprendizaje^(12,15–19)^. Estas simulaciones permiten a los participantes asumir el protagonismo del escenario, ejerciendo la toma de decisiones de forma segura en un entorno controlado, sin exposición a pacientes reales, preservando así su integridad física, así como situaciones relacionadas con la ética profesional cotidiana^(13,15–18)^.

Por lo tanto, se cree que la implementación de este tipo de simulación virtual tiene el potencial de mejorar los resultados del aprendizaje y, en consecuencia, promover la seguridad del paciente, ayudando a cambiar actitudes y comportamientos a través de la formación con simulación virtual. También cabe destacar la posibilidad de desarrollar habilidades técnicas y no técnicas, generalmente consistentes en procedimientos propios de cada especialidad, a cualquier nivel de profundidad. Las habilidades no técnicas implican las competencias cognitivas y sociales que complementan a las técnicas para que el profesional pueda ejercer con calidad y seguridad, imprescindibles para que las enfermeras puedan trabajar en escenarios reales de cuidados críticos^(28)^.

Desde esta perspectiva, se entiende que la implementación de simulaciones basadas en escenarios ramificados permite a los participantes interactuar con diferentes personajes del equipo de salud y pacientes, discutir las situaciones presentadas en los casos, tomar decisiones basadas en el razonamiento clínico y, consecuentemente, aprender las mejores prácticas. Sin embargo, cabe destacar que para que esta actividad se desarrolle de forma efectiva, es necesario construir una guía clínica detallada, que contenga los objetivos de aprendizaje y las habilidades a desarrollar por los participantes en los escenarios propuestos.

El escenario de simulación virtual fue desarrollado como una oportunidad para proporcionar un ambiente seguro de enseñanza-aprendizaje, a un menor costo y aumentando la posibilidad de participación, a través del acceso online en cualquier momento o lugar, desde dispositivos móviles o fijos. La construcción de escenarios de simulación clínica es un proceso cuidadoso que debe ser desarrollado con base en evidencias científicas, además de ser sometido a revisión por pares con experiencia clínica en el área y a pruebas piloto con el público objetivo. Por lo tanto, esta tarea de desarrollo y actualización es cíclica y debe ir acompañada de una retroalimentación continua a los participantes de la simulación^(15–19)^.

Durante el desarrollo se prestó especial atención a la interfaz de la simulación virtual desarrollada, que se define como el medio a través del cual el usuario puede comunicarse con un determinado sistema informatizado para llevar a cabo una determinada actividad. Su finalidad es promover una interacción amistosa entre los seres humanos y las máquinas, por medio de elementos visualizados en la pantalla^(29,30)^.

En este estudio, las etapas de los escenarios ramificados fueron descritas y producidas con diferentes profesionales para garantizar la eficacia de la tecnología educativa y con vistas a que esta tecnología pueda apoyar a las enfermeras en los cuidados requeridos en la ENP.

## CONCLUSIÓN

La utilización de buenas prácticas para el desarrollo del escenario de simulación virtual ramificado ha llevado a reflexiones sobre los cuidados prestados a los pacientes de la UCI, vinculadas a la seguridad del paciente, especialmente en la prevención de eventos adversos graves como la ENP.

Los resultados de las evaluaciones realizadas por los jueces validaron el contenido y el aspecto de la tecnología, y sugirieron cambios que se tendrán en cuenta antes de su plena implantación. Entre las limitaciones del estudio figuran el tiempo necesario para completar todas las fases previstas y el reducido tamaño de la muestra.

Los puntos fuertes destacados por los jueces que evaluaron la tecnología fueron la búsqueda de nuevos objetivos y retos de aprendizaje y la importancia de esta tecnología innovadora para su uso en la formación y educación continua de enfermeras profesionales, que también podría aplicarse a la formación de estudiantes de enfermería de pregrado.

Como perspectiva para futuros estudios, la integración de modelos lingüísticos a gran escala (LLM), como ChatGPT^®^, será una oportunidad para aportar nuevas dimensiones a la experiencia educativa de los enfermeros. Estos modelos podrían utilizarse para generar automáticamente diálogos realistas entre personajes en escenarios de simulación, proporcionando a los participantes una interacción más auténtica que simule con precisión los matices de la comunicación humana en contextos clínicos. Además, podrían servir de asistentes virtuales durante las simulaciones, ofreciendo orientación clínica basada en pruebas e información inmediata sobre las decisiones tomadas por los estudiantes. Esto no sólo aumentaría el realismo de las simulaciones virtuales, sino que también permitiría una forma de formación escalable e interactiva, que se adaptaría en tiempo real a las respuestas de los participantes y a los caminos elegidos en los escenarios ramificados. El potencial de personalización del aprendizaje a partir de este tipo de simulación virtual es considerable, permitiendo simulaciones que se adaptan al nivel de experiencia del profesional, reforzando la adquisición de conocimientos y el desarrollo de competencias críticas. Por tanto, el uso de los LLM abre un campo prometedor para enriquecer la educación en salud y ampliar los límites de la formación clínica interactiva.

## DISPONIBILIDAD DE DATOS

Los datos están disponibles en el repositorio FIGSHARE^®^: https://doi.org/10.6084/m9.figshare.24463573.v1.
